# Five Complete *Salmonella enterica* Serotype Reading Genomes Recovered from Patients in the United States

**DOI:** 10.1128/mra.00388-22

**Published:** 2022-06-21

**Authors:** Hattie E. Webb, Kaitlin A. Tagg, Justin Y. Kim, Elizabeth A. Miller, Timothy J. Johnson, Arancha Peñil-Celis, Fernando de la Cruz, Jason P. Folster

**Affiliations:** a ASRT, Inc, Atlanta, Georgia, USA; b Division of Foodborne, Waterborne, and Environmental Diseases, Centers for Disease Control and Prevention, Atlanta, Georgia, USA; c Department of Veterinary and Biomedical Sciences, College of Veterinary Medicine, University of Minnesota, Saint Paul, Minnesota, USA; d Instituto de Biomedicina y Biotecnología de Cantabria, Universidad de Cantabria, Santander, Spain; University of Maryland School of Medicine

## Abstract

Between 2018 and 2019, Salmonella enterica serotype Reading caused a large, multistate outbreak linked to contact with raw turkey products in the United States. Here, we provide five Salmonella Reading reference genomes collected from US patients between 2016 and 2018.

## ANNOUNCEMENT

Salmonella enterica serotype Reading is uncommonly associated with human illness, but caused a multistate outbreak linked to contact with raw turkey products from 2018 to 2019 in the US (1). Previous phylogenetic analyses identified three Hadar clades. Clade 1 contained the “emergent” 2018 to 2019 outbreak-associated subclade, which was genetically distinct from a “contemporary” subclade of previously circulating Reading strains, partially due to the acquisition of mobile genetic elements (MGE) (2). Clade 2 primarily contained human isolates, and its pangenome was considerably smaller than those of the other clades, but further analysis to understand these differences was not performed (2). Closed sequences of Reading are required for pangenome exploration and to further understand the novel “emergent” subclade, but only 11 are currently available (3, 4), and none are from US patients. Here, we generated five complete clinical Reading sequences from Clades 1 and 2 to serve as references.

Five Reading isolates from human illnesses were chosen for long-read sequencing to represent human-associated Reading diversity before and during the outbreak: two “contemporary” Clade 1 isolates (2018), one “emergent” Clade 1 isolate (2017), and two Clade 2 isolates collected before the outbreak (2016 to 2017). Isolates originated from clinical diagnostic or public health laboratories (PHL) as part of the CDC’s national passive Salmonella surveillance (https://www.cdc.gov/nationalsurveillance/salmonella-surveillance.html); thus, isolation methods vary by site (5). Serotype was confirmed *in silico* using SeqSero2 v0.1 (6). Genomic DNA was extracted (Wizard Genomic DNA purification kit, modified manufacturer’s protocol, Promega, WI, USA) from cultures incubated on tryptic soy agar-sheep blood overnight (37°C). Libraries were prepared (Rapid Barcoding kit SQK-RBK004; manufacturer’s protocol, Oxford Nanopore Technologies [ONT], Oxford, United Kingdom) and sequenced for 72 h on a GridION sequencing platform (R9.4.1 flowcells; ONT). Reads were base-called using Guppy v4.2.2 and filtered for quality using MinKNOW (ONT). Hybrid assemblies were generated, polished, circularized, and rotated using Unicycler v0.4.8 (conservative option) (7); the corresponding Illumina short reads (previously generated at PHL through PulseNet, https://www.cdc.gov/pulsenet/) were accessed through NCBI’s Short Read Archive (https://www.ncbi.nlm.nih.gov/sra). Assemblies were quality controlled using QUAST v5.0.2 (8) and blastn v2.9.0 (9). Resistance determinants and plasmid replicons were detected using an internal workflow that employs the ResFinder database (downloaded 30JUL2020; 90% identity, 50% coverage) and the PointFinder scheme for Salmonella spp. (downloaded 30 August 2019), and an in-house database adapted from PlasmidFinder (90% identity, 60% coverage; https://cge.food.dtu.dk/services/PlasmidFinder/), all implemented in staramr v.0.4.0 (https://github.com/phac-nml/staramr). Plasmid taxonomic units (PTUs) were identified using COPLA (10). Sequence types (ST) were determined using staramr (multilocus sequence typing [MLST] software [https://github.com/tseemann/mlst] and the PubMLST database [11]). The default parameters were used for all software unless otherwise specified.

Consistent with the previous analysis (2), Clade 1 genomes were larger than Clade 2 genomes by at least ~110 kb, due to the presence of plasmids and MGE (Table 1). Of note, PNUSAS014950 contained a ~10-kb resistance plasmid (replicons ColpHAD28 and Col440II, PTU not assigned; Table 1) that was previously found to be significantly more common in the “emergent” 2018 to 2019 outbreak-associated subclade (2). This plasmid was first seen in Reading in 2014 and may be of particular interest for further investigation (2).

**TABLE 1:** Summary information for five Salmonella enterica serotype Reading genomes isolated from humans in the US^*a*^

Strain no.	NCBI accession numbers				Short-read		Long-read					GC content (%)	Total size (bp)	Collection yr	Clade^*b*^	ST	Resistance determinants	Plasmid replicon(s)	PTU
	BioSample	Short read SRA	GenBank	Long read SRA	Mean read length	No. of reads	Contig *N*_50_ (bp)	*N*_50_ (bp)	Mean read length (bp)	No. of reads	No. of contigs								
PNUSAS060563	SAMN10601908	SRR8327737	CP093147, CP093148	SRR18753113	241	835,744	4,678,719	10,579	5,144.1	208,391	2	52.19	4,680,815	2018	Clade 1 contemporary	412	None	ColpVC	PTU-E11
PNUSAS039653	SAMN09199050	SRR7155940	CP093129, CP093130, CP093131	SRR18753114	245	1,043,912	4,681,904	11,665	5,454	201,613	3	52.06	4,742,074	2018	Clade 1 contemporary	412	None	IncI2(delta), ColpVC	PTU-I2, PTU-E11
PNUSAS014950	SAMN07193435	SRR5659649	CP093132, CP093133	SRR18753116	241	556,070	4,647,387	11,386	4,975.5	148,565	2	52.20	4,657,771	2017	Clade 1 emergent	412	*bla*_TEM-1C_	ColpHAD28, Col440II	No PTU assigned^*c*^
PNUSAS003019	SAMN05437761	SRR3979113	CP093134	SRR18753117	240	1,431,150	4,543,956	12,425	5,968.3	284,196	1	52.19	4,543,956	2016	2	93	None	None	None
PNUSAS020177	SAMN07436240	SRR19599933	CP094293	SRR18753115	255	4,123,842	4,544,675	13,652	6,436.9	146,777	1	52.19	4,544,696	2017	2	93	None	None	None

a ST, sequence type; PTU, plasmid taxonomic unit.

b Whole-genome core single nucleotide polymorphism (SNP)-based phylogenetic clade as defined by Miller et al. (2).

c No PTU assigned, but query is part of a sHSBM cluster of size 2 using COPLA (https://castillo.dicom.unican.es/copla/ [10]).

### Data availability.

The sequences discussed here have been deposited in GenBank and SRA under the accession and BioSample numbers listed in Table 1.

## References

[1] Hassan R, Buuck S, Noveroske D, Medus C, Sorenson A, Laurent J, Rotstein D, Schlater L, Freiman J, Douris A, Simmons M, Donovan D, Henderson J, Tewell M, Snyder K, Oni O, Von Stein D, Dassie K, Leeper M, Adediran A, Dowell N, Gieraltowski L, Basler C. 2019. Multistate outbreak of *Salmonella* infections linked to raw turkey products: United States, 2017–2019. MMWR Morb Mortal Wkly Rep 68:1045–1049. doi:10.15585/mmwr.mm6846a1. 31751325 PMC6871895

[2] Miller EA, Elnekave E, Flores-Figueroa C, Johnson A, Kearney A, Munoz-Aguayo J, Tagg KA, Tschetter L, Weber BP, Nadon CA, Boxrud D, Singer RS, Folster JP, Johnson TJ. 2020. Emergence of a novel *Salmonella enterica* serotype Reading clonal group is linked to its expansion in commercial Turkey production, resulting in unanticipated human illness in North America. MSphere 5:e00056-20. doi:10.1128/mSphere.00056-20. 32295868 PMC7160679

[3] Li C, Tyson GH, Hsu C-H, Harrison L, Strain E, Tran T-T, Tillman GE, Dessai U, McDermott PF, Zhao S. 2021. Long-read sequencing reveals evolution and acquisition of antimicrobial resistance and virulence genes in *Salmonella enterica*. Front Microbiol 12:777817. doi:10.3389/fmicb.2021.777817. 34867920 PMC8640207

[4] Zhao S, Li C, Hsu C-H, Tyson GH, Strain E, Tate H, Tran T-T, Abbott J, McDermott PF. 2020. Comparative genomic analysis of 450 strains of *Salmonella enterica* isolated from diseased animals. Genes 11:1025. doi:10.3390/genes11091025. PMC7564550 32883017

[5] Tolar B, Joseph LA, Schroeder MN, Stroika S, Ribot EM, Hise KB, Gerner-Smidt P. 2019. An overview of PulseNet USA databases. Foodborne Pathog Dis 16:457–462. doi:10.1089/fpd.2019.2637. 31066584 PMC6653802

[6] Zhang S, den Bakker HC, Li S, Chen J, Dinsmore BA, Lane C, Lauer AC, Fields PI, Deng X. 2019. SeqSero2: rapid and improved *Salmonella* serotype determination using whole-genome sequencing data. Appl Environ Microbiol 85:e01746-19. doi:10.1128/AEM.01746-19. 31540993 PMC6856333

[7] Wick RR, Judd LM, Gorrie CL, Holt KE. 2017. Unicycler: resolving bacterial genome assemblies from short and long sequencing reads. PLoS Comput Biol 13:e1005595. doi:10.1371/journal.pcbi.1005595. 28594827 PMC5481147

[8] Mikheenko A, Prjibelski A, Saveliev V, Antipov D, Gurevich A. 2018. Versatile genome assembly evaluation with QUAST-LG. Bioinformatics 34:i142–i50. doi:10.1093/bioinformatics/bty266. 29949969 PMC6022658

[9] Altschul SF, Gish W, Miller W, Myers EW, Lipman DJ. 1990. Basic Local Alignment Search Tool. J Mol Biol 215:403–410. doi:10.1016/S0022-2836(05)80360-2. 2231712

[10] Redondo-Salvo S, Bartomeus-Peñalver R, Vielva L, Tagg KA, Webb HE, Fernández-López R, de la Cruz F. 2021. COPLA, a taxonomic classifier of plasmids. BMC Bioinformatics 22:390. doi:10.1186/s12859-021-04299-x. 34332528 PMC8325299

[11] Jolley KA, Maiden MC. 2010. BIGSdb: scalable analysis of bacterial genome variation at the population level. BMC Bioinformatics 11:595. doi:10.1186/1471-2105-11-595. 21143983 PMC3004885

